# A Hardy Plant Facilitates Nitrogen Removal via Microbial Communities in Subsurface Flow Constructed Wetlands in Winter

**DOI:** 10.1038/srep33600

**Published:** 2016-09-20

**Authors:** Penghe Wang, Hui Zhang, Jie Zuo, Dehua Zhao, Xiangxu Zou, Zhengjie Zhu, Nasreen Jeelani, Xin Leng, Shuqing An

**Affiliations:** 1 School of Life Science and Institute of Wetland Ecology, Nanjing University, Nanjing, P. R. China; 2 Nanjing University Ecology Research Institute of Changshu (NJUecoRICH), Changshu, P. R. China

## Abstract

The plants effect in subsurface flow constructed wetlands (SSF-CWs) is controversial, especially at low temperatures. Consequently, several SSF-CWs planted with *Iris pseudacorus* (CW_I_) or *Typha orientalis* Presl. (CW_T_) and several unplanted ones (CW_C_) were set up and fed with secondary effluent of sewage treatment plant during the winter in Eastern China. The 16S rDNA Illumina Miseq sequencing analysis indicated the positive effects of *I*. *pseudacorus* on the bacterial community richness and diversity in the substrate. Moreover, the community compositions of the bacteria involved with denitrification presented a significant difference in the three systems. Additionally, higher relative abundances of nitrifying bacteria (0.4140%, 0.2402% and 0.4318% for *Nitrosomonas*, *Nitrosospira* and *Nitrospira*, respectively) were recorded in CW_I_ compared with CW_T_ (0.2074%, 0.0648% and 0.0181%, respectively) and CW_C_ (0.3013%, 0.1107% and 0.1185%, respectively). Meanwhile, the average removal rates of NH_4_^+^-N and TN in CW_I_ showed a prominent advantage compared to CW_C_, but no distinct advantage was found in CW_T_. The hardy plant *I*. *pseudacorus*, which still had active root oxygen release in cold temperatures, positively affected the abundance of nitrifying bacteria in the substrate, and accordingly was supposed to contribute to a comparatively high nitrogen removal efficiency of the system during the winter.

During the past several decades, constructed wetlands (CWs), which are an economical and environmentally friendly wastewater treatment technology, have been widely used for removing pollutants from a variety of wastewaters including domestic wastewater, agricultural wastewater, industrial wastewater, acid mine drainage, etc. around the world^1^,^2^,^3^,^4^,^5^. They are generally categorized into two basic types: free water surface system (FWS-CWs) and subsurface flow system (SSF-CWs). In recent years, SSF-CWs have attracted more and more studies and applications in China and Europe, due to its decreased demand for land and high efficiency for various pollutants^1^. Researches on the mechanism of pollutants removal in CWs have revealed that it is a fairly complex process which involves a variety of removal pathways, including microbial degradation, plant uptake, substrate filtration and adsorption, precipitation, sedimentation and volatilization^3^,^6^,^7^,^8^.

Microorganisms have been universally realized as an original and critical factor in the wetland purification process^8^,^9^,^10^. Biodegradation of organic compounds in wastewater is primarily due to some heterotrophic and autotrophic bacteria, for example, methane oxidizing bacteria (methanotrophs) and sulfate-reducing bacteria (SRB). Moreover, nitrification-denitrification and anaerobic ammonium oxidation (anammox) are usually considered to be the two key links for nitrogen removal from wastewater in wetlands. Ammonia oxidizing bacteria (AOB), mainly belonging to *β-proteobacteria* and *γ-proteobacteria*, determined the rate-limiting step for nitrification that converts ammonium to nitrite with oxygen as the final electron acceptor, while a wider variety of bacterial phylogenetic groups contributed to the denitrification process^8^,^11^.

Plants have several properties that make them an essential component in wetlands, but their exact effect is complex and controversial. Apart from absorbing and storing the nutrients from wastewater, the presence of macrophytes is believed to have a close relationship with the increased microbial density, activity and diversity in wetlands^6^,^7^,^12^,^13^. On the one hand, plant roots can provide a substratum for attached microorganism growth^1^,^8^. On the other hand, the plant litter can serve as carbon source for heterotrophic bacteria^6^,^13^,^14^. In addition, the emerging plants growing in SSF-CWs can also work as oxygen transfer passage for aerobic microorganism in the substrate^6^,^15^,^16^. However, several studies posited that plants had little statistical effect on the community abundance or structure of the overall bacteria or particular microbial functional groups^17^,^18^,^19^,^20^. In fact, the interaction between the macrophyte and microorganism community depends on a variety of factors such as plant species, vegetation growth status, season and even random chance^17^,^20^,^21^,^22^. Therefore, the effects of different plants on microorganism groups in CWs, especially in the cold season, need deeper exploration. Further study on the complicated process occurring in CWs would be beneficial to optimizing design parameters and improving the overall performance in engineering application practices.

In the present research, yellow flag (*Iris pseudacorus*) and oriental cattail (*Typha orientalis* Presl.) were chosen. The two different species have different physiological properties and growth characteristics; for example, the yellow flag can remain active, but the acrial part of the oriental cattail will wither away in the winter. Furthermore, to investigate the microorganism community in the substrate, the V3-V4 regions of the bacteria 16S rDNA were sequenced via an Illumina MiSeq 2500 platform. Because of its higher integrity and broader range of applications^23^,^24^, 16S rDNA Illumina Miseq sequencing has recently been used frequently to study the microbial diversity in various environments^25^,^26^,^27^,^28^. Recently, this technology is also used for the analysis of microorganism communities in CWs^29^,^30^,^31^,^32^,^33^,^34^.

## Results

### Overall performance

**Figure 1:**
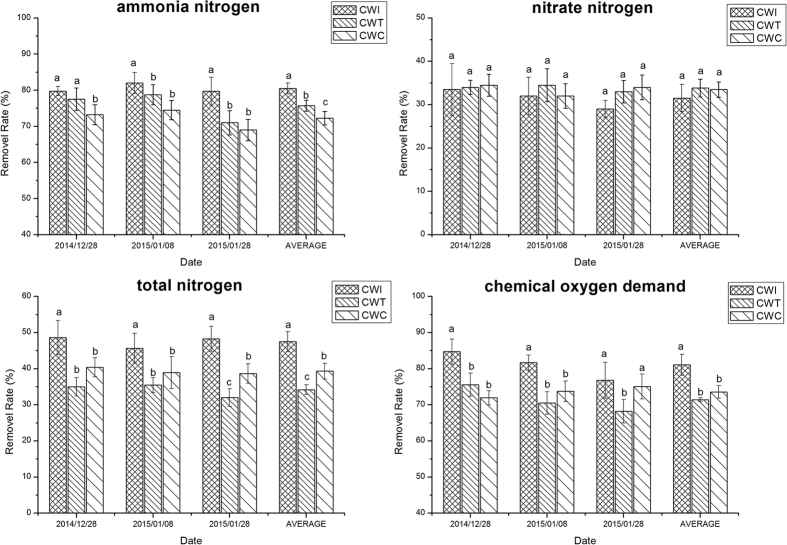
Removal efficiencies (%) of ammonia nitrogen (NH_4_^+^-N), nitrate nitrogen (NO_3_^−^-N), total nitrogen (TN) and chemical oxygen demand (COD) from the three types of constructed wetlands during the winter (n = 4). Different letters indicate significant differences (p < 0.05) among the types of constructed wetlands on the same date.

The influent characteristics and the time for water sample determination were described in the second section (Experimental Design and Operation). The overall performance of the two types of SSF-CWs is shown in Fig. 1. CW_I_ had the highest removal rates of NH_4_^+^-N (80.50%), TN (47.50%) and COD (81.07%) and was superior to the control, which had removal rates of 72.25% (NH_4_^+^-N), 39.33% (TN) and 73.57% (COD), respectively. For CW_T_, only NH_4_^+^-N removal (75.75%) presented a slight advantage compared to the control, while the removal rates of TN (34.17%) and COD (71.43%) were the lowest among the three systems. The removal efficiency of NO_3_^−^-N in the three systems was consistent at approximately 33.00%. NO_2_^−^-N was not detected in the effluent.

### Growth and physiological characteristics of the plants

Plant biomasses and nitrogen accumulations in the plants were determined during Stage III of the experiment (Table 1). The dry weight of the *I*. *pseudacorus* shoot increased slightly from 226.75 gDW·m^−2^ to 249.35 gDW·m^−2^, although no significant difference was observed. Conversely, the biomass of the *T*. *orientalis* Presl. shoot declined sharply from 135.24 gDW·m^−2^ to 50.91 gDW·m^−2^ for the dormancy of aboveground part. However, for the roots biomasses, both of species rose indistinctively. The *I*. *pseudacorus* shoot and *T*. *orientalis* Presl. shoot increased from 633.47 gDW·m^−2^ to 681.83 gDW·m^−2^ and 552.31 gDW·m^−2^ to 562.86 gDW·m^−2^, respectively. With the change in the plant biomasses, the nitrogen accumulation in the plants changed accordingly. An increase of 0.42 g·m^−2^, 0.38 g·m^−2^ and 0.06 g·m^−2^ and a decrease of 0.46 g·m^−2^ were recorded for the *I*. *pseudacorus* shoot, *I*. *pseudacorus* root, *T*. *orientalis* Presl. root and *T*. *orientalis* Presl. shoot, respectively. The rate of root radial oxygen loss (ROL) and the root vitality in the two plants were also measured (Table 1). The rate of ROL in *I*. *pseudacorus* was 5.95 μmolO_2_·g^−1^Root·h^−1^ and showed a striking difference compared to the *T*. *orientalis* Presl., which recorded as 1.73 μmolO_2_·g^−1^Root·h^−1^. Similarly, the root vitality of the *I*. *pseudacorus* root was 347.48 μgTTC·g^−1^Root·h^−1^ while it was 56.44 μgTTC·g^−1^Root·h^−1^ in the *T*. *orientalis* Presl. root.

### Richness and diversity of the microbial communities

Three 16S rDNA libraries were established based on the Miseq Illumina sequencing of the three microorganism samples (Table 2). With 95% similarity, 4732, 3209 and 2991 Operational Taxonomic Units (OTUs) were clustered with a Good’s Coverage of 0.96, 0.97 and 0.98 in CW_I_, CW_T_ and CW_C_, respectively. The total numbers of OTUs estimated by the ACE estimator were 8550, 7527 and 5401. The Shannon indexes were 6.57, 5.75 and 5.36 in CW_I_, CW_T_ and CW_C_, respectively. With 97% similarity, 10393, 9118 and 7475 OTUs were clustered with a Good’s Coverage of 0.91, 0.90 and 0.92 respectively. The ACE estimators were 34531.54, 29659.38, 25433.24, and the Shannon indexes were 7.83, 7.87 and 7.28, respectively.

### Microbial community composition

**Figure 2:**
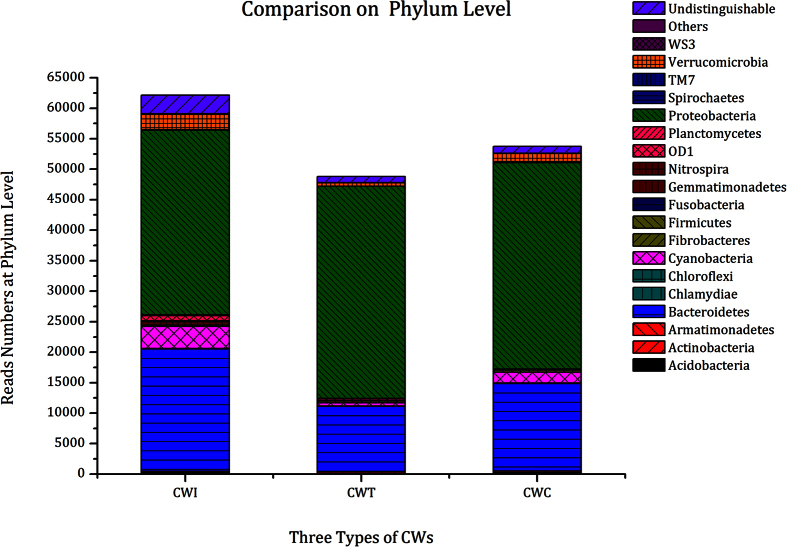
The bacterial communities in CW_I_, CW_T_ and CW_C_ at the phylum level. Some Phyla (reads numbers < 10) are grouped into “Others”.

In addition to the richness and diversity of the microbial communities, the community composition in the substrate also plays a critical role in the pollutants removal in CWs. The abundance of different phyla of bacteria in the three types of SSF-CWs is shown in Fig. 2. A total of 24 identifiable phyla, in which *Proteobacteria* and *Bacteroidetes* were the two dominant species, were detected in the three systems. In CW_I_, *Proteobacteria* and *Bacteroidetes* accounted for 51.16% and 33.91%, respectively, followed by *Cyanobacteria* (6.05%) and *Verrucomicrobia* (4.23%). In CW_T_, *Proteobacteria* and *Bacteroidetes* accounted for 72.73% and 22.59%, respectively. In the control, *Proteobacteria* (64.29%) and *Bacteroidetes* (27.26%) constituted the primary phyla, and *Cyanobacteria* (3.39%) and *Verrucomicrobia* (2.55%) followed.

**Figure 3:**
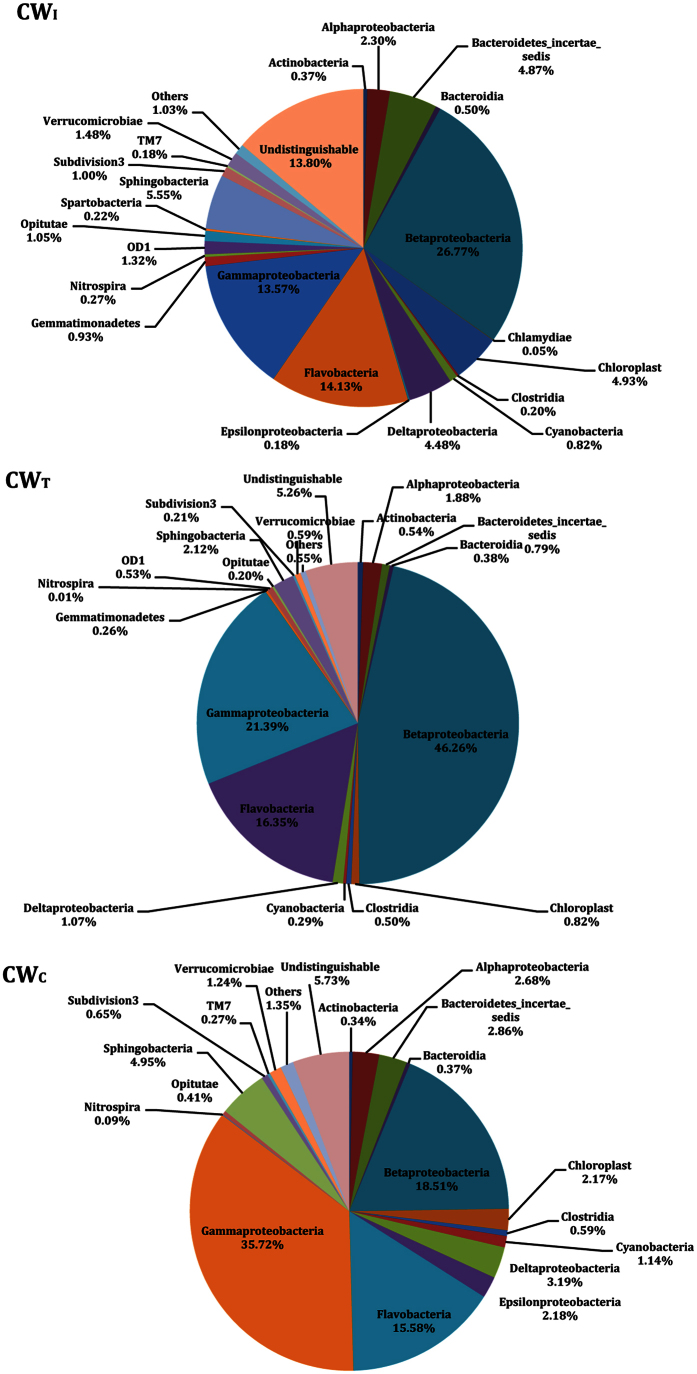
The relative abundances of the bacterial communities in CW_I_, CW_T_ and CW_C_ at the class level. Some classes (relative abundances < 0.15%, except for *Nitrospira*) are grouped into “Others”.

The bacterial composition of the three systems at the class level is shown in Fig. 3. Overall, 52, 42 and 50 classes were observed, with 13.80%, 5.26% and 5.71% of the total reads in each sample being undistinguishable at the present taxonomic level in CW_I_, CW_T_ and CW_C_, respectively. The order of the primary classes was *β-proteobacteria* (26.77%) > *Flavobacteria* (14.13%) > *γ-proteobacteria* (13.57%) > *Sphingobacteria* (5.55%) > *Chloroplast* (4.93%) > *δ-proteobacteria* (4.48%) > *α-proteobacteria* (2.30%) in CW_I_. In CW_T_, it was *β-proteobacteria* (46.26%) > *γ-proteobacteria* (21.39%) > *Flavobacteria* (16.35%) > *Sphingobacteria* (2.12%) > *α-proteobacteria* (1.88%) > *δ-proteobacteria* (1.07%). In CW_C_, they mainly included *γ-proteobacteria* (35.72%), *β-proteobacteria* (18.51%), *Flavobacteria* (15.58%), *Sphingobacteria* (4.95%), *δ-proteobacteria* (3.91%), *α-proteobacteria* (2.68%) and *ε-proteobacteria* (2.18%).

The primary genera (relative abundance >0.50%) with the addition of three nitrifying bacteria (*Nitrosomonas*, *Nitrosospira* and *Nitrospira*) in the three systems are shown in Table 3. A total of 40 genera were listed, and they mainly belonged to two classes, *β-proteobacteria* (17 genera) and *γ-proteobacteria* (10 genera). The bacterial community composition showed a significant difference at the genera level among the three systems. In CW_I_, *Flavobacterium* (18.28%), *Albidiferax* (10.02%) and *Ohtaekwangia* (7.72%) constituted the three dominant genera. In CW_T_, they were *Deefgea* (22.21%), *Flavobacterium* (19.47%), *Albidiferax* (14.08%) and *Halomonas* (12.21%). In CW_C_, the two dominant genera composed of *Halomonas* (27.81%) and *Flavobacterium* (17.62%). Among the 40 listed genera, there were at least 8 genera reported to involve in denitrification. They included *Azospira*, *Dechloromonas*, *Aeromonas*, *Shewanella*, *Halomonas*, *Pseudomonas*, *Arcobacter* and *Flavobacterium*. The compositions of these denitrifying bacteria were also different among the three systems. In CW_I_, the order of their relative abundances was *Flavobacterium* (18.28%) > *Halomonas* (2.91%) > *Aeromonas* (1.81%) > *Pseudomonas* (1.68%) > *Dechloromonas* (1.29%) > *Azospira* (0.67%) > *Shewanella* (0.56%) > *Arcobacter* (0.24%). In CW_T_, there were richer *Flavobacterium* (19.47%) and *Halomonas* (12.21%). In CW_C_, the dominant denitrifying bacteria were *Halomonas* (27.81%) and *Flavobacterium* (17.62%), followed by *Aeromonas* (3.22%) and *Arcobacter* (2.99%). In addition, *Nitrosomonas*, *Nitrosospira* and *Nitrospira* were also observed in the three systems, although their relative abundances were not rich. CW_I_ presented the highest proportion of nitrifying bacteria, and the relative abundances were 0.4140% (*Nitrosomonas*), 0.2402% (*Nitrosospira*) and 0.4318% (*Nitrospira*), respectively. The lowest percentage of the three nitrifiers was observed in CW_T_, which recorded as 0.2074% (*Nitrosomonas*), 0.0648% (*Nitrosospira*) and 0.0181% (*Nitrospira*), respectively. In CW_C_, their abundances were 0.3013% (*Nitrosomonas*), 0.1107% (*Nitrosospira*) and 0.1185% (*Nitrospira*), respectively.

## Discussions

It was widely acknowledged that the transformation and removal of nitrogen in CWs were mainly attributed to nitrification and denitrification, followed by vegetation assimilation, substrate adsorption and the other pathways^3^,^4^,^6^,^7^,^8^,^35^. Because the sorption of ammonia by gravel or sand was not the primary means for ammonia removal^22^, only the nitrogen contents in the water and plants were determined in this research. Our results showed that the nitrogen accumulation in the vegetation was very limited during the variables determination stage (Stage III). This result was related to the weak growth of *I*. *pseudacorus* and the dormant aboveground part of *T*. *orientalis* during the cold season. Before the variable determination, the current systems had operated normally for 6 months, which was considered a long enough time for the microbial community in the substrate to reach a steady state^13^,^36^,^37^. Compared to traditional molecular methods, 16S rDNA Illumina Miseq sequencing is a more effective approach for studying microbial diversity due to its higher throughput and integrity as well as its broader application range^23^,^24^. PCR-DGGE is often restricted by an insufficient resolution to characterize the microbial communities in the complex samples^13^, while 454-pyrosequencing has a low throughput and high error rate and is costly^24^. In the present study, approximately ten thousand OTUs were identified in each sample, which were clustered with 97% similarity. This result was 4–5 times higher than that in several previous studies on this issue^22^,^38^.

Many researchers supported that the microorganism composition was closely related to the vegetable species in wetlands^15^,^17^,^21^,^22^,^35^,^39^. To reveal if there are any significant differences in the bacterial communities among the three types of CWs in the present research, a detailed comparison was carried out at different taxonomic levels. Firstly, the results indicated that *Proteobacteria* was the dominant phyla followed by *Bacteroidetes* in the three systems. This finding was consistent with a plenty of studies that reported *Proteobacteria* as the dominant phylum in various microorganism samples from the substrate or rhizosphere in wetlands^22^,^38^,^40^,^41^. *Proteobacteria*, which displayed a remarkably high level of bacterial metabolic diversity involved in global carbon, nitrogen and sulfur cycling, played an important role in pollutants removal^40^,^42^. Further analysis at the class level showed an obvious difference in bacterial community compositions among the three systems. The bacterial community mainly consisted of *β-proteobacteria*, *Flavobacteria* and *γ-proteobacteria* in CW_I_, while it was highly enriched with *β-proteobacteria* in CW_T_ and the dominant class was *γ-proteobacteria* in CW_C_. Due to the amount of different influential factors, such as wetland types, substrate characteristics, wastewater components, operational parameters, environmental conditions and the different estimations by different approaches used in previous CW studies, the dominant class in CWs has been a long debated subject^13^,^43^,^44^. The present results suggested that the different plants with different growth characteristics and physiological properties had a considerable effect on the bacterial community compositions in CWs.

According to the list for genera in which at least one member had been characterized as a denitrifying strain reported by Heylen, *et al*.^45^ and Philippot, *et al*.^46^, it was found that a considerable proportion of the bacterial genera detected in the present research had a close relationship to denitrification. The compositions of denitrifying bacteria presented a remarkable difference among the three systems. The system grown with a hardy plant showed superior evenness of denitrifying bacteria, while certain genus such as *Flavobacterium* or *Halomonas* presented a fairly high abundance in the freezing-sensitive plant system and the unplanted system. However, it seemed that all the three types of SSF-CWs had adequate denitrification capability.

The difference in the overall efficiency of nitrogen removal among the three systems might result from the nitrification process. The bacteria involved in nitrification mainly consisted of two groups, the ammonium-oxidizing bacteria (AOB), which converted ammonium to nitrite, and the nitrite-oxidizing bacteria (NOB), which converted nitrite to nitrate^11^. A higher ratio of NOB to AOB populations was considered indicative of a higher nitrification capacity and more complete ammonia oxidation^47^. In this research, higher relative abundance of nitrifying bacteria and higher ratio of NOB to AOB populations accompanied by higher removal efficiency of NH_4_^+^-N were recorded in CW_I_ compared to CW_T_ and CW_C_. This phenomenon could be attributed to more oxygen being released from the hardy plant *I*. *pseudacorus*^6^,^15^. Zhong, *et al*.^11^ also reported that enhanced levels of oxygen and nitrite favoured the growth of NOB in CWs. In the present experiment, *Nitrosomonas* and *Nitrosospira* were the two dominant AOB lineages. This finding was consistent with a previous study in a vertical flow CW by Tietz, *et al*.^48^. This study also found that *Nitrosomonas* in the three systems was more abundant than *Nitrosospira*. The result might be related to the high influent NH_4_^+^-N concentration, which contributed to the formation of a community dominated by *Nitrosomonas*, because *Nitrosomonas* had a lower substrate affinity but a higher maximum activity than *Nitrosospira*^49^,^50^. Moreover, *Nitrospira* was the only NOB detected in the present systems. These results suggested that the three nitrifying bacteria, *Nitrosomonas*, *Nitrosospira* and *Nitrospira*, shared the most responsibility for nitrification.

The diversity of the microbial composition suggested a diversity of the functional characteristics that were relevant to pollutant removal in wastewater^51^. Any difference in microbial functional groups might lead directly to the difference in the purification performance of the CWs^11^. Alpha diversity analysis suggested that the presence of a hardy plant induced higher diversity and evenness of bacterial community composition at different taxonomic levels ranging from class to genus. This might be related to the multiple influencing mechanisms of plants on microorganisms such as complicating the attachment surface, providing organic carbon, as well as changing the oxidation-reduction conditions in the wetlands^1^,^6^,^15^. The results revealed by the ACE estimator and Shannon index indicated that the system grown with hardy plant had higher microbial richness. This finding was consistent with several earlier studies^22^,^38^,^52^,^53^. The oxygen released from plant roots could affect the microorganism richness in the substrate^39^. Previous studies have shown that the bacterial communities were grouped according to an oxygen concentration gradient^40^,^54^,^55^. In the current study, the two plants grown in the CWs have different physiological properties and growth characteristics. During the experiment period, *I*. *pseudacorus* remained active, but the acrial part of *T*. *orientalis* withered away. The rate of root radial oxygen loss (ROL) was also higher in CW_I_ compared to CW_T_. This might contribute to a higher richness of aerobic microorganism in CW_I_. Moreover, the carbon source supply is also an important factor that influences the microbial communities in the substrate^6^,^8^. The senescent or dead plant could release a variety of labile organic compounds such as amino acids, sugars, volatile fatty acids, etc.^56^. In this research, *T*. *orientalis*, a dormant plant during the winter, showed a decline in shoot biomass because of the senescence of leaves during the determination period. Chen *et al*.^38^,^57^,^58^ suggested that the enhanced litter decomposition might contribute to increased microbial richness in the substrate of the CW. However, in this study, the effect of plant litter on denitrification efficiency enhancement was limited in the *T*. *orientalis* system. This could be because quite a large proportion of plant litter, such as highly crystalline lignocellulose, was recalcitrant and the complete degradation of litter needed a relatively long time^7^,^59^. Therefore, the present results suggested a positive effect of the hardy plant on the nitrogen removal in SSF-CWs during the winter, while the freezing-sensitive plant showed slight differences on the pollutants removal compared to the control.

In addition, some other factors may also have significant impact on the contaminants removal in CWs. Plant root exudates can also be used as the dissolved organic matter for the metabolism of heterotrophic bacteria^13^. In the current research, due to the limitations of experimental conditions, the root vitality was measured as a proxy indicator. The results seem to indicate that the presence of *I*. *pseudacorus* have more active effects towards the microbial community compositions in the substrate. Moreover, the root activity and growth status of the macrophytes in CWs also influence the richness and enzyme activity of microbial populations^10^,^52^,^60^. Wang, *et al*.^61^ reported that plant ROL played an important role in nutrient removal by significantly affecting microbial abundance and activity in CWs. Additionally, anammox was also recognized as another approach for nitrogen removal apart from nitrification-denitrification^62^. However, the anammox bacteria, placed in the order *Brocadiales* in the phylum *Planctomycetes*, has not been observed in this study. Besides, the coexistence of some autotrophic bacteria such as *Cyanobacteria* in wetlands was also reported benefit to the pollutants degradation, because they could provide the heterotrophic bacteria with oxygen, which played a similar and complementary role in addition to the macrophytes to a certain degree^22^,^63^. Furthermore, some archaea with specific activity of ammonia oxidation also play an indispensable role in nitrogen removal processes^64^,^65^. All these factors should be taken into consideration in further researches.

In conclusion, 16S rDNA Illumina Miseq sequencing revealed detailed information about the interaction between the plant and the bacterial community composition in SSF-CWs. The hardy plant *I*. *pseudacorus* had a positive effect on the bacterial abundance and eventually on the community composition in the substrate. The presence of a hardy plant with active root oxygen release enhanced the relative abundance of nitrifying bacteria in the substrate, and consequently was supposed to contribute to the increase in the nitrogen removal efficiency of the system during the winter. However, some other factors, such as the bacteria population and activity, the presence of *Cyanobacteria* and ammonia-oxidizing archaea may also have an important influence on the nitrogen removal in SSF-CWs. Hence, further researches will be needed to understand detailed mechanisms referring to the biodegradation process of pollutants in CWs.

## Methods

### Experimental Design and Operation

To simulate subsurface flow constructed wetlands (SSF-CWs), four outdoor mesocosms were planted with *I*. *pseudacorus*, four were planted with *T*. *orientalis* Presl., both at an initial density of 30 plants per m^2^, and four controls without plants were built in Huai’an, Jiangsu province, China (33.3°N, 119.0°E). The dimensions of each mesocosm were (0.8 m)^2^ × π × 0.75 m, and each bed consisted of three layers: 100 mm deep rough sand (1–2 mm in diameter) on the top, 100 mm deep gravel (10–20 mm in diameter, porosity of 0.45) in the middle, and 550 mm deep gravel (30–50 mm in diameter, porosity of 0.55) at the bottom (Fig. S1).

The operation of the SSF-CWs was divided into three stages. (1) Stage I (From June 10^th^, 2014 to Sept 10^th^, 2014) was for plants growth. The mesocosms were fed continuously with natural water until the plant shoots grew long enough. (2) Stage II (From Sept 10^th^, 2014 to Dec 10^th^, 2014) was for microbial establishment. The mesocosms were fed continuously with wastewater with appropriate artificial modification on the base of a secondary effluent from a sewage treatment plant for domestic wastewater. The pH of the influent water was 7.12–7.88, the average hydraulic loading rate (HLR) was 187.5 mm·d^−1^ and the hydraulic retention time (HRT) was 4 days. The concentrations of dissolved oxygen (DO), chemical oxygen demand (COD), ammonia nitrogen (NH_4_^+^-N), nitrite nitrogen (NO_2_^−^-N), nitrate nitrogen (NO_3_^−^-N) and total nitrogen (TN) in the influent water were 9.2–9.8 mg·L^−1^, 55.0–65.0 mg·L^−1^, 9.5–12.5 mg·L^−1^, 0.3–0.6 mg·L^−1^, 4.5–5.5 mg·L^−1^ and 18.0–24.0 mg·L^−1^, respectively. (3) Stage III (From Dec 10^th^, 2014 to Feb 10^th^, 2015) was for variables determination. The operation scheme was the same as Stage II. The water temperature was 4.3–6.7 °C during Stage III.

### Water Sampling and Analysis

The water temperature in the mesocosms was recorded by the Temperature and Illuminance Data Logger (HOBO Pendant UA-002-08, Onset, USA). The pH was determined by a portable Multi-parameter Water Quality Meter (U-52, HORIBA, Japan). Dissolved oxygen (DO) was monitored *in situ* using DO electrodes (HQ40d-53 LED, HACH, USA). The concentrations of NH_4_^+^-N, NO_2_^−^-N, NO_3_^−^-N, TN and COD were determined with a Water Quality Analyzing System (DRB200 and DR2800, HACH, USA). All variables were analysed according to standard analytical procedures^66^.

### Plant Sampling and Analysis

Plant samples were harvested and separated into roots and shoots, dried at 65 °C to a constant weight, and then ground into powder. The N content was determined by an Elemental analyzer (CHN-O-Rapid, Heraeus, Germany) at the beginning and end of the experiment^67^. The rate of root radial oxygen loss (ROL) was measured using the titanium (III) citrate buffer method^68^,^69^. The root vitality was quantified via the triphenyl tetrazolium chloride (TTC) method^70^.

### Microbial Sampling and Analysis

#### Preparation of microbial samples

As shown in Fig. S1, microorganism sampling points of each system was divided into three layers according to the substrate. Each layer comprised four sampling points. The four points formed a circle with a radius of 0.4 m around the system center, and the connections between each point and the system center formed a cross. 100 g of sand from the top layer and 100 g of gravel from the middle layer were obtained by a cylindrical sampler with 2.5 cm diameter, while 200 g of gravel were obtained from the bottom layer by a sampling scoop on Feb 2^nd^ 2015. Then, the samples in each system were mixed well and vigorously shaken at 200 rpm for 3 h in sterile glass bottles to isolate the biofilm from the substrate surface. After centrifuging twice (6,000 × g, 15 min), the precipitate was collected for subsequent DNA extraction, 16S rDNA PCR amplification and Illumina MiSeq sequencing.

#### DNA extraction and PCR amplification

DNA was extracted from the samples using a QIAamp Fast DNA Stool Mini Kit (QIAGEN, Chatsworth, CA, USA) according to the manufacturer’s instructions. DNA yields were determined using a SpectraMax 190 (Molecular Devices, California, USA), and the integrity was evaluated via 1.0% agarose gel electrophoresis. Then, DNA was diluted to 1 ng·μL^−1^ in sterile water. The universal primer sets 341F (5′-CCTAYGGGRBGCASCAG-3′) and 785R (5′-GACTACHVGGGTATCTAATCC-3′) were used for amplification of the V3-V4 regions of 16S rDNA. A 10 ng template, 0.5 μL of forward primers and 0.5 μL of reverse primers were added into a 25 μL reaction system for the PCR reaction. Thermal cycling consisted of denaturation at 94 °C for 3 min, which was followed by 30 cycles of 94 °C for 10 s, 55 °C for 15 s and 72 °C for 30 s, and finally held at 72 °C for 7 min. AmpureBeads (Beckman Coulter, Inc., CA, USA) were used for the PCR product purification.

#### 16S rDNA Illumina MiSeq sequencing

The sequencing libraries were constructed with an NEB Next Ultra DNA Library Prep Kit for Illumina (New England Biolabs Inc., Boston, MA, USA), and then a Qubit 2.0 Fluorometer (Life Invitrogen, Inc., Carlsbad, CA, USA) was used to assess the libraries quality. Then, the libraries were sequenced on an Illumina MiSeq 2500 platform.

#### Quality filtering, OTUs picking, annotation and diversity analysis

The FASTX-Toolkit (version 0.0.14) and Mothur program (version 1.34.0) were used for the analysis, merging and quality filtering of the raw data sequences. All of the reads were quality filtered using an average quality value of 20 (Q20) during demultiplexing. Short reads (length < 40 bp) and chimeras were excluded. Reads were clustered by degree similarity levels with the UCLUST program (version 1.2.22q). Sequences with ≥ 97% similarity were assigned to the same genus. A RDP classifier (version 2.2) was used to annotate the taxonomic information. The Mothur program was used for alpha diversity analysis. ACE estimated the richness of the species, while the Shannon index estimated the diversity of the species.

### Statistical Analysis

All statistical tests were performed with the statistical program SPSS 17.0 (SPSS Inc. Chicago, USA). The data were analysed with one-way analysis of variance to compare the performance of each mesocosm. Duncan’s test was performed to detect the statistical significance of differences (p > 0.05) between the mean values of the treatments.

## Additional Information

**How to cite this article**: Wang, P. *et al*. A Hardy Plant Facilitates Nitrogen Removal via Microbial Communities in Subsurface Flow Constructed Wetlands in Winter. *Sci. Rep*. **6**, 33600; doi: 10.1038/srep33600 (2016).

## Supplementary Material

Supplementary Information

Supplementary Table S1

## Figures and Tables

**Table 1:** Biomass, nitrogen accumulation, ROL rate and root activity in *I*. *pseudacorus* CW and *T*. *orientalis* Presl. CW (means ± std., *p* < 0.05, n = 10).

	*I. pseudacorus*		*T. orientalis* Presl.	
	Shoot	Root	Shoot	Root
Initiative biomass (gDW·m^−2^)	226.75 ± 15.32	633.47 ± 51.55	135.24 ± 3.57	552.31 ± 51.57
Terminative biomass (gDW·m^−2^)	249.35 ± 22.32	681.83 ± 51.55	50.91 ± 2.31	562.86 ± 53.12
Increased biomass (gDW·m^−2^)	22.60	48.36	−84.33	10.55
N Percentage (%)	1.85 ± 0.016	0.78 ± 0.0065	0.54 ± 0.0044	0.53 ± 0.0038
N Accumulation (g·m^−2^)	0.42	0.38	−0.46	0.06
ROL rate (μmolO_2_·g^−1^Root·h^−1^)	\	5.95 ± 1.34	\	1.73 ± 0.28
Root activity (μgTTC·g^−1^Root·h^−1^)	\	347.48 ± 14.22	\	56.44 ± 5.71

**Table 2:** Comparison of phylotype coverage and diversity estimation of the 16S rRNA gene libraries at the 3% dissimilarity from the Miseq high-throughput sequencing analysis.

	Samples	OTUs	ACE	Shannon	Coverage
α = 0.03	***I***. ***pseudacorus***	10393	34531.54	7.83	0.91
	***T***. ***orientalis*** **Presl**.	9118	29659.38	7.87	0.90
	**The Control**	7475	25433.24	7.28	0.92
α = 0.05	***I***. ***pseudacorus***	4732	8549.64	6.57	0.96
	***T***. ***orientalis*** **Presl**.	3209	7527.14	5.75	0.97
	**The Control**	2991	5401.41	5.36	0.98

The coverage (Good’s coverage), richness estimators (ACE) and diversity indices (Shannon) were calculated using the Mothur program.

**Table 3:** The main genera (relative abundance >0.50%) with the addition of three nitrifying bacteria (*Nitrosomonas*, *Nitrosospira* and *Nitrospira*) in CW_I_, CW_T_ and CW_C_.

Class	Order	Family	Genus	Reads numbers			Relative abundances (%)		
				CW_I_	CW_T_	CW_C_	CW_I_	CW_T_	CW_C_
*β-proteobacteria*	*Burkholderiales*	*Burkholderiaceae*	*Polynucleobacter*	175	544	222	0.45	1.41	0.57
*β-proteobacteria*	*Burkholderiales*	*Comamonadaceae*	*Albidiferax*	**3921**	**5433**	**1118**	**10**.**02**	**14**.**08**	**2**.**88**
*β-proteobacteria*	*Burkholderiales*	*Comamonadaceae*	*Aquabacterium*	196	13	13	0.50	0.03	0.03
*β-proteobacteria*	*Burkholderiales*	*Comamonadaceae*	*Hydrogenophaga*	257	49	190	0.66	0.13	0.49
*β-proteobacteria*	*Burkholderiales*	*Comamonadaceae*	*Limnohabitans*	23	196	8	0.06	0.51	0.02
*β-proteobacteria*	*Burkholderiales*	*Comamonadaceae*	*Malikia*	22	458	16	0.06	1.19	0.04
*β-proteobacteria*	*Burkholderiales*	*Comamonadaceae*	*Polaromonas*	229	213	221	0.59	0.55	0.57
*β-proteobacteria*	*Burkholderiales*	*Oxalobacteraceae*	*Janthinobacterium*	105	367	102	0.27	0.95	0.26
*β-proteobacteria*	*Methylophilales*	*Methylophilaceae*	*Methylophilus*	419	142	193	1.07	0.37	0.50
*β-proteobacteria*	*Neisseriales*	*Neisseriaceae*	*Chitinibacter*	645	**1358**	**1575**	1.65	**3**.**52**	**4**.**06**
*β-proteobacteria*	*Neisseriales*	*Neisseriaceae*	*Deefgea*	**1604**	**8567**	**790**	**4**.**10**	**22**.**21**	**2**.**03**
*β-proteobacteria*	*Neisseriales*	*Neisseriaceae*	*Iodobacter*	162	261	117	0.41	0.68	0.30
*β-proteobacteria*	*Rhodocyclales*	*Rhodocyclaceae*	*Azospira*	264	41	104	0.67	0.11	0.27
*β-proteobacteria*	*Rhodocyclales*	*Rhodocyclaceae*	*Dechloromonas*	503	80	177	1.29	0.21	0.46
*β-proteobacteria*	*Rhodocyclales*	*Rhodocyclaceae*	*Sulfuritalea*	388	146	115	0.99	0.38	0.30
*γ-proteobacteria*	*Aeromonadales*	*Aeromonadaceae*	*Aeromonas*	709	**876**	**1249**	1.81	**2**.**27**	**3**.**22**
*γ-proteobacteria*	*Aeromonadales*	*Aeromonadaceae*	*Tolumonas*	**1700**	**771**	108	**4**.**34**	**2**.**00**	0.28
*γ-proteobacteria*	*Alteromonadales*	*Alteromonadaceae*	*Haliea*	203	31	243	0.52	0.08	0.63
*γ-proteobacteria*	*Alteromonadales*	*Psychromonadaceae*	*Psychromonas*	6	522	114	0.02	1.35	0.29
*γ-proteobacteria*	*Alteromonadales*	*Shewanellaceae*	*Shewanella*	219	137	**929**	0.56	0.36	**2**.**39**
*γ-proteobacteria*	*Chromatiales*	*Chromatiaceae*	*Rheinheimera*	206	232	507	0.53	0.60	1.31
*γ-proteobacteria*	*Enterobacteriales*	*Enterobacteriaceae*	*Serratia*	701	**1134**	718	1.79	**2**.**94**	1.85
*γ-proteobacteria*	*Oceanospirillales*	*Halomonadaceae*	*Halomonas*	**1138**	**4709**	**10800**	**2**.**91**	**12**.**21**	**27**.**81**
*γ-proteobacteria*	*Pseudomonadales*	*Pseudomonadaceae*	*Cellvibrio*	288	110	702	0.74	0.29	1.81
*γ-proteobacteria*	*Pseudomonadales*	*Pseudomonadaceae*	*Pseudomonas*	659	**793**	**1029**	1.68	**2**.**06**	**2**.**65**
*δ-proteobacteria*	*Bdellovibrionales*	*Bacteriovoracaceae*	*Bacteriovorax*	**1019**	208	**1355**	**2**.**60**	0.54	**3**.**49**
*δ-proteobacteria*	*Bdellovibrionales*	*Bacteriovoracaceae*	*Peredibacter*	**1076**	136	194	**2**.**75**	0.35	0.50
*ε-proteobacteria*	*Campylobacterales*	*Campylobacteraceae*	*Arcobacter*	94	25	**1161**	0.24	0.06	**2**.**99**
*Flavobacteria*	*Flavobacteriales*	*Cryomorphaceae*	*Algoriphagus*	**810**	154	519	**2**.**07**	0.40	1.34
*Flavobacteria*	*Flavobacteriales*	*Cryomorphaceae*	*Fluviicola*	460	174	297	1.18	0.45	0.76
*Flavobacteria*	*Flavobacteriales*	*Flavobacteriaceae*	*Flavobacterium*	**7152**	**7509**	**6844**	**18**.**28**	**19**.**47**	**17**.**62**
*Flavobacteria*	*Flavobacteriales*	*Flavobacteriaceae*	*Luteolibacter*	338	137	389	0.86	0.36	1.00
*Sphingobacteria*	*Sphingobacteriales*	*Saprospiraceae*	*Haliscomenobacter*	199	35	90	0.51	0.09	0.23
uncertain	uncertain	uncertain	*Ohtaekwangia*	**3023**	382	**1538**	**7**.**72**	0.99	**3**.**96**
*Gemmatimonadetes*	*Gemmatimonadales*	*Gemmatimonadaceae*	*Gemmatimonas*	579	126	43	1.48	0.33	0.11
*Verrucomicrobiae*	*Opitutales*	*Opitutaceae*	*Opitutus*	630	95	212	1.61	0.25	0.55
*Verrucomicrobiae*	*Verrucomicrobiales*	*Verrucomicrobiaceae*	*Prosthecobacter*	329	84	81	0.84	0.22	0.21
*β-proteobacteria*	*Nitrosomonadales*	*Nitrosomonadaceae*	*Nitrosomonas*	162	80	117	0.4140	0.2074	0.3013
*β-proteobacteria*	*Nitrosomonadales*	*Nitrosomonadaceae*	*Nitrosospira*	94	25	43	0.2402	0.0648	0.1107
*Nitrospira*	*Nitrospirales*	*Nitrospiraceae*	*Nitrospira*	169	7	46	0.4318	0.0181	0.1185

Primary genera (relative abundance >2.00%) in each sample were bolded.
