# Supplementary material for: A Hardy Plant Facilitates Nitrogen Removal via Microbial Communities in Subsurface Flow Constructed Wetlands in Winter
Source: Sci Rep. 2016 Sep 20;6:33600. doi: 10.1038/srep33600 (PMC5028706; doi:10.1038/srep33600)

A Hardy Plant Facilitates Nitrogen Removal via Microbial Communities in Subsurface Flow Constructed Wetlands in Winter

Penghe Wang^,2^, Hui Zhang^1^, Jie Zuo^1^, Dehua Zhao^1^*, Xiangxu Zou^1^, Zhengjie Zhu^1,2^, Nasreen Jeelani^1^, Xin Leng^1,2^*, Shuqing An^1,2^

1 School of Life Science and Institute of Wetland Ecology, Nanjing University, Nanjing, P. R. China

2 Nanjing University Ecology Research Institute of Changshu (NJUecoRICH), Changshu, P. R. China

**Fig. S1** The SSF-CW system and the microbial sampling points.


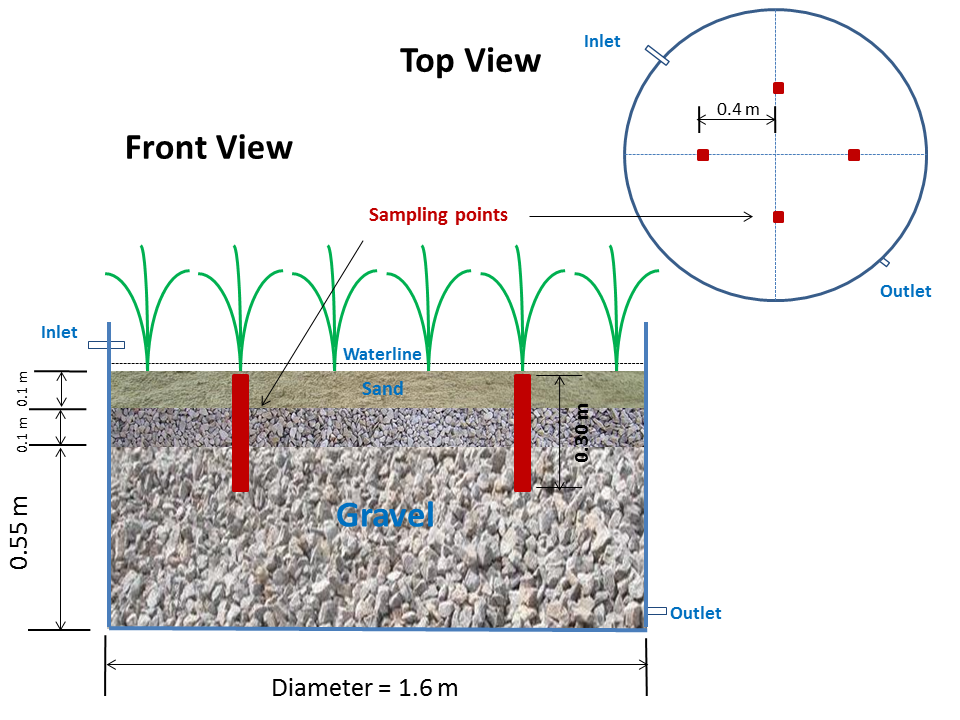

Supplement: Supplementary Information [file srep33600-s1.doc]
