# Supplementary material for: A Hardy Plant Facilitates Nitrogen Removal via Microbial Communities in Subsurface Flow Constructed Wetlands in Winter
Source: Sci Rep. 2016 Sep 20;6:33600. doi: 10.1038/srep33600 (PMC5028706; doi:10.1038/srep33600)
Supplement: Supplementary Table S1 [file srep33600-s2.doc]

A Hardy Plant Facilitates Nitrogen Removal via Microbial Communities in Subsurface Flow Constructed Wetlands in Winter

Penghe Wang^,2^, Hui Zhang^1^, Jie Zuo^1^, Dehua Zhao^1^*, Xiangxu Zou^1^, Zhengjie Zhu^1,2^, Nasreen Jeelani^1^, Xin Leng^1,2^*, Shuqing An^1,2^

1 School of Life Science and Institute of Wetland Ecology, Nanjing University, Nanjing, P. R. China

2 Nanjing University Ecology Research Institute of Changshu (NJUecoRICH), Changshu, P. R. China

**Table S1** The concentrations of chemical oxygen demand (COD) (before and after filtration) and total nitrogen (TN) (before and after filtration) from the effluent water of the three CWs and the influent water (n = 4).

|  | **COD before filtration** | **COD after filtration** | **TN before filtration** | **TN after filtration** |
| --- | --- | --- | --- | --- |
| **CW_I_ 1** | 12.20 | 10.40 | 12.39 | 11.2 |
| **CW_I_ 2** | 12.00 | 10.50 | 11.13 | 9.87 |
| **CW_I_ 3** | 9.80 | 9.10 | 10.36 | 9.68 |
| **CW_I_ 4** | 10.00 | 8.70 | 11.76 | 9.2 |
| **Mean** | 11.00 | 9.68 | 11.41 | 9.99 |
| **Std.** | 1.28 | 0.91 | 0.87 | 0.86 |
|  |  |  |  |  |
| **CW_T_ 1** | 18.40 | 16.30 | 14.07 | 12.3 |
| **CW_T_ 2** | 16.40 | 14.90 | 13.44 | 11.7 |
| **CW_T_ 3** | 16.00 | 14.60 | 13.02 | 12.1 |
| **CW_T_ 4** | 20.00 | 18.20 | 13.65 | 12.5 |
| **Mean** | 17.70 | 16.00 | 13.55 | 12.15 |
| **Std.** | 1.86 | 1.64 | 0.44 | 0.34 |
|  |  |  |  |  |
| **CW_C_ 1** | 16.00 | 14.30 | 13.86 | 12.3 |
| **CW_C_ 2** | 18.00 | 16.40 | 13.30 | 12.1 |
| **CW_C_ 3** | 14.00 | 12.60 | 12.39 | 11.4 |
| **CW_C_ 4** | 15.00 | 13.70 | 11.76 | 10.2 |
| **Mean** | 15.75 | 14.25 | 12.83 | 11.50 |
| **Std.** | 1.71 | 1.60 | 0.93 | 0.95 |
|  |  |  |  |  |
| **Inlet 1** | 60.20 | 57.20 | 22.30 | 20.1 |
| **Inlet 2** | 64.40 | 61.20 | 22.10 | 20.4 |
| **Inlet 3** | 57.90 | 55.30 | 20.30 | 18.8 |
| **Inlet 4** | 59.30 | 55.90 | 22.90 | 20.5 |
| **Mean** | 60.45 | 57.40 | 21.90 | 19.95 |
| **Std.** | 2.80 | 2.65 | 1.12 | 0.79 |
